# Disruption of the LRRK2 substrate RAB12 facilitates neurotransmission and causes hyperactivity in mice

**DOI:** 10.1038/s41531-026-01353-4

**Published:** 2026-04-24

**Authors:** Xingjian Li, Yuanxin Chen, Huaixing Wang, Xue Zhang, Noah Guy Lewis Guiberson, Xianting Li, Jacqueline Burré, Junmin Peng, Hui Zhang, Zhenyu Yue

**Affiliations:** 1 Department of Neurology, Friedman Brain Institute, Icahn School of Medicine at Mount Sinai, New York, NY USA; 2 Department of Physiology & Pharmacology, College of Veterinary Medicine, The University of Georgia, Athens, GA USA; 3 Department of Neuroscience, Thomas Jefferson University, Philadelphia, PA USA; 4 Departments of Structural Biology and Developmental Neurobiology, St. Jude Children’s Research Hospital, Memphis, TN USA; 5 Brain and Mind Research Institute, Appel Alzheimer’s Disease Research Institute, Weill Cornell Medicine, New York, NY USA; 6 Department of Neuroscience, Friedman Brain Institute, Icahn School of Medicine at Mount Sinai, New York, NY USA; 7 Center for Parkinson’s Disease Neurobiology, Friedman Brain Institute, Icahn School of Medicine at Mount Sinai, New York, NY USA

**Keywords:** Cell biology, Neuroscience

## Abstract

RAB12 is a small GTPase and a validated substrate of LRRK2, a kinase genetically linked to Parkinson’s disease (PD). While RAB12–LRRK2 signaling has been implicated in ciliogenesis and immune regulation, the neuronal function of RAB12 remains largely unexplored. Here, we investigated the role of RAB12 in synaptic physiology using *Rab12* knockout (KO) mice. *Rab12* KO mice developed normally but exhibited increased locomotor activity in adulthood. Electrophysiological recordings from striatal slices revealed enhanced presynaptic release probability and increased excitatory drive onto medium spiny neurons. Consistently, live-cell imaging of cultured cortical neurons revealed that *Rab12* deletion facilitated, while *Rab12* overexpression inhibited, synaptic vesicle exocytosis. Biochemical fractionation showed enrichment of RAB12 in synaptic vesicle–associated fractions containing presynaptic components. Proteomic analysis of *Rab12* KO striatal synaptosomes further identified alterations in proteins involved in synaptic membrane trafficking pathways. Together, these findings establish RAB12 as a negative regulator of synaptic vesicle exocytosis and excitatory neurotransmission in vivo. Our study defines a physiological role for RAB12 in synaptic function and provides a basis for future investigation into how LRRK2-dependent RAB12 signaling may contribute to neuronal dysfunction in PD.

## Introduction

Small GTPase RAB proteins are crucial for an array of neuronal functions, particularly vesicular trafficking, and their dysfunction can contribute to neurodegenerative diseases. A subset of Rab GTPases, including RAB12, is phosphorylated by leucine-rich repeat kinase 2 (LRRK2)^1^, genetic variants of which are linked to common familial cases of Parkinson’s disease (PD). LRRK2 regulates various cellular pathways^2^, including presynaptic vesicle trafficking in neurons^3–5^. RAB12 interacts with effectors such as RILP1, RILPL2, and RILP to regulate secretion^6^, autophagy^7^, ciliogenesis^8^, and endosomal-lysosomal degradation^9^. RAB12 promotes microtubule-associated retrograde transport of secretory granules, thereby negatively regulating their release in mast cells^6^, which suggests a role in vesicle secretion. Additionally, RAB12 promotes autophagosome trafficking via interaction with LC3^7^ and coordinates with the clathrin adapter complex-1 to sort the epidermal growth factor receptor from the trans-Golgi network^10^. Despite the importance of RAB12 in vesicular trafficking, its function in neurons, particularly in synaptic vesicle (SV) trafficking, remains unclear.

Our previous study and others have identified RAB12 as a robust LRRK2 substrate in the mouse brain^1,11,12^. RAB12 acts as an LRRK2 activator, enhances LRRK2’s activity^13^, and facilitates its lysosomal recruitment^14^. Moreover, RAB12 regulates ciliogenesis and centrosome homeostasis in astrocytes^11^, and deletion of *Rab12* rescues ciliary deficiency and centrosome cohesion defects caused by the PD-associated LRRK2 G2019S mutation in astrocytes. Cryo-EM analysis demonstrated that RAB12 and LRRK2 form a complex, and that the RAB12–LRRK2 interaction is required for RAB12 function in ciliogenesis and centrosome regulation in astrocytes^11^.

We and others previously showed that LRRK2 variants deregulate SV trafficking^3–5^. Given the structural and functional relationship between RAB12 and LRRK2, we investigated whether RAB12 plays a role in SV trafficking in neurons. Here, we characterized *Rab12* knock-out (KO) mice using behavioral tests, electrophysiology, live-cell imaging, biochemical, and molecular biology approaches, and identified a role of RAB12 in regulating SV trafficking.

## Results

### Locomotor hyperactivity in *Rab12* KO mice

**Fig. 1:**
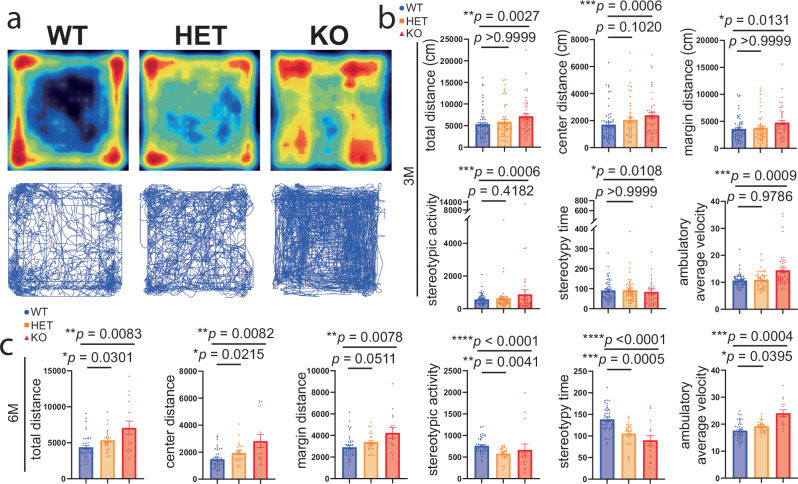
Behavioral analysis of *Rab12* KO mice. **a** Representative heat maps and path tracings from the open field test of WT, *Rab12*^+/−^ (HET), and *Rab12*^*−/−*^ (KO) mice at 3 months of age. **a**, **b** Quantification of behavioral assessments in the open field test for WT, *Rab12*^+/−^, and *Rab12*^*−/−*^ mice at 3 months (**b**) and 6 months (**c**) of age. Data from three different cohorts, including both male and female mice, were combined for statistical analysis. Each data point in the graphs represents one mouse. At 3 months: *n*_WT_ = 60, *n*_HET_ = 47, and *n*_KO_ = 47. At 6 months: *n*_WT_ = 42, *n*_HET_ = 24, and *n*_KO_ = 15. Outliers were identified using the ROUT method (*Q* = 1%). All outliers are shown in the graphs, whereas statistical analyses were performed after outlier removal. The Kruskal–Wallis test followed by the Dunn’s multiple comparisons test was used for (**b**, **c**), except for ambulatory average velocity in (**c**), which was analyzed using the Welch’s ANOVA followed by the Dunnett’s T3 multiple comparisons test. *p* values are indicated in the graphs. *p < 0.05; **p < 0.01; ***p < 0.001; ****p < 0.0001. Error bars represent the SEM.

We previously established the *Rab12* KO (*Rab12*^−/−^) mouse line^11^. Mutant mice with heterozygous (+/−) and homozygous (−/−) deletion of *Rab12* developed normally, with no differences in body weight in adulthood (3 and 6 months) (Fig. S1a). In the open field test, however, *Rab12*^−/−^ mice exhibited significantly greater total, center, and margin movement distances, along with higher ambulatory average velocity, at 3 months (Fig. 1a, b) and 6 months (Fig. 1c). In contrast, *Rab12*^+/−^ mice displayed few changes compared to control mice. Notably, *Rab12*^−/−^ mice showed reduced stereotypic activity and stereotypic time at both 3 and 6 months (Fig. 1a–c), whereas *Rab12*^+/−^ mice showed this reduction only at 6 months (Fig. 1a–c). We noticed a sex-dependent difference in the age of onset of specific behavioral features during the open field test. For example, at 3 months, *Rab12*^−/−^ male mice exhibited decreased stereotypic activity and stereotypic time, and increased ambulatory velocity, but did not show changes in total, center, and margin movement distances compared to controls. *Rab12*^−/−^ female mice displayed elevated total, center, and margin movement distances, with little change in stereotypy or ambulatory velocity (Fig. S1b). At 6 months, *Rab12*^−/−^ males showed increased total, center, and margin movement distances and ambulatory velocity, but decreased stereotypy, whereas *Rab12*^−/−^ females exhibited decreased stereotypy and elevated ambulatory velocity but no alterations in movement distances (Fig. S1c). Taken together, our findings indicate that both male and female *Rab12*^−/−^ mice display hyperactive movement while exhibiting reduced repetitive motor behaviors.

Furthermore, we observed no difference in the rotarod test between mutant and control mice (Fig. S1d). Interestingly, *Rab12*^−/−^ mice displayed a moderate but significant enhancement of grip strength at 3 months (Fig. S1e, left panel), whereas this difference diminished by 6 months (Fig. S1e, right panel).

### Enhanced medium spiny neuron excitability and synaptic transmission in *Rab12* KO mice

**Fig. 2:**
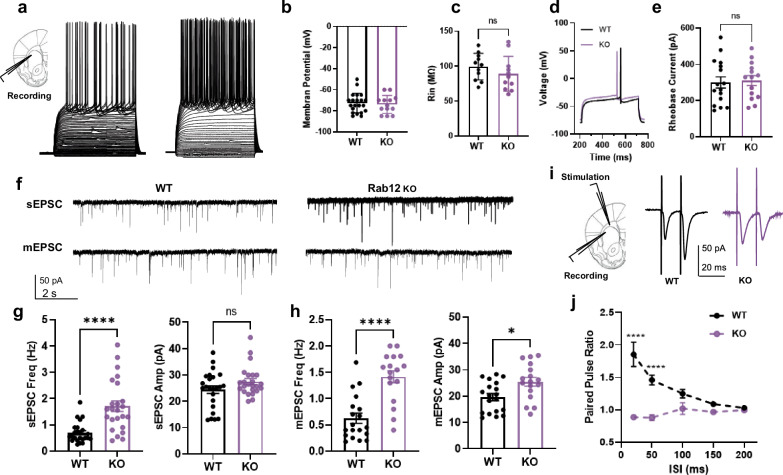
Electrophysiological recording of medium spiny neurons (MSNs) of the dorsal striatum. **a** Schematic showing the coronal section used for placing recording electrodes in the dorsal striatum (left), and representative current-clamp traces of MSNs from WT and *Rab12* KO mice in response to depolarizing current steps (right). **b**–**e** Intrinsic electrophysiological properties of MSNs: resting membrane potential, input resistance, and rheobase current, were not significantly altered in *Rab12* KO mice, with (**d**) showing the representative trace of the first action potential elicited at rheobase current inject in WT and *Rab12* KO MSNs (Mann–Whitney test; resting membrane potential: *p* = 07186; input resistance: *p* = 0.9287; rheobase: *p* = 0.7198). **f** Representative traces of spontaneous excitatory postsynaptic currents (sEPSCs, top) and miniature EPSCs (mEPSCs, bottom) recorded from MSNs of WT and *Rab12* KO mice at a holding potential of −80 mV. **g** sEPSC frequency was significantly increased in *Rab12* KO mice, while the amplitude remained unchanged (Mann–Whitney test; frequency: ****p < 0.0001; amplitude: *p* = 0.2702). **h** Both the frequency and the amplitude of mEPSCs were significantly elevated in *Rab12* KO MSNs compared to WT, suggesting alterations in both presynaptic release and postsynaptic responsiveness (Mann–Whitney test; frequency: ****p < 0.0001; amplitude: *p* = 0.0131). **i** Left: Schematic indicating stimulation and recording electrode placement in paired-pulse experiments. Right: Representative paired-pulse EPSC traces recorded from MSNs of WT and *Rab12* KO mice. **j** Paired-pulse ratios (EPSC2/EPSC1) were significantly reduced at interstimulus intervals (ISIs) of 20 ms and 50 ms in *Rab12* KO MSNs, indicating increased presynaptic release probability (Two-way ANOVA, *F* (1, 63) = 73.45), ****p < 0.0001, 20 ms: ****p < 0.0001; 50 ms: ****p < 0.0001; 100 ms: *p* = 0.0963; 150 ms: *p* = 0.7335; 200 ms: *p* = 0.9988).

Striatal medium spiny neurons (MSNs) express high levels of LRRK2^15^ and play a critical role in motor control^16–18^. To understand the neuronal function of RAB12, we focused on MSNs and examined their intrinsic electrophysiological properties in *Rab12*^−/−^ mice. We performed whole-cell current-clamp recordings from MSNs in the dorsal striatal slices of WT and *Rab12*^−/−^ mice (Fig. 2a). MSNs from *Rab12*^−/−^ animals exhibited more action potentials in response to depolarizing current injection compared to WT controls. However, the resting membrane potential, input resistance, and rheobase current were not significantly different from WT controls (Fig. 2b–e), suggesting that intrinsic excitability was largely preserved.

To assess the impact of *Rab12* on spontaneous synaptic activity, we recorded spontaneous excitatory postsynaptic currents (sEPSCs) from MSNs (Fig. 2f). *Rab12*^−/−^ mice displayed a significant increase in sEPSC frequency, with no change in amplitude (Fig. 2g), suggesting enhanced excitatory input. In the presence of TTX, both the frequency and amplitude of mEPSCs were elevated in *Rab12*^−/−^ neurons (Fig. 2h), indicating increased presynaptic release probability and possible postsynaptic receptor upregulation.

To investigate the functional connectivity of cortical inputs, we applied paired-pulse stimulation to the corpus callosum and recorded evoked responses in MSNs (Fig. 2i), with interstimulus intervals (ISIs) of 20, 50, 100, 150, and 200 ms. The pair-pulse ratio (PPR) was used to assess presynaptic release probability. A significant decrease in PPR at 20 and 50 ms ISIs was observed in *Rab12*^−/−^ mice relative to WT controls (Fig. 2j), consistent with increased presynaptic glutamate release from cortical afferents.

Collectively, these results demonstrate that *Rab12*^−/−^ leads to enhanced excitatory neurotransmission in the dorsal striatal MSNs, implicating RAB12 as a negative regulator of synaptic strength in the dorsal striatum.

### Regulation of synaptic vesicle exocytosis by RAB12 in cortical neurons

**Fig. 3:**
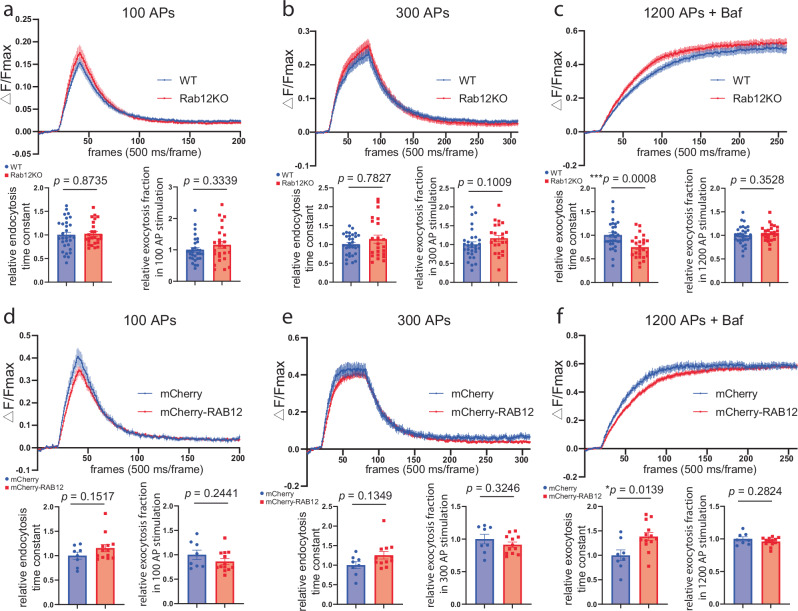
Live-cell imaging of presynaptic trafficking in *Rab12* KO and *Rab12*-overexpressing neurons. pHluorin assays were performed to assess synaptic vesicle endocytosis and exocytosis in primary cortical neurons from WT and *Rab12* KO mice (**a**–**c**), and in primary cortical neurons overexpressing mCherry or mCherry-RAB12 (**d**–**f**). Average pHluorin traces were obtained from neurons stimulated with 100 action potentials (APs; 10 Hz for 10 s; **a**, **d**), 300 APs (10 Hz for 30 s; **b**, **e**), or 1200 APs (10 Hz for 120 s) in the presence of bafilomycin (Baf; **c**, **f**). Cells in (**a**–**c**) were derived from five neonatal WT mice and five neonatal *Rab12* KO mice from four independent cultures, whereas cells in (**d**–**f**) were obtained from four neonatal WT mice from two independent cultures. Each data point represents one neuron (**a**: *n*_WT_ = 30, *n*_Rab12KO_ = 25; **b**: *n*_WT_ = 30, *n*_Rab12KO_ = 23; **c**: n_WT_ = 31, *n*_Rab12KO_ = 25; **d**, **f**: *n*_mCherry_ = 8, *n*_mCherry-RAB12_ = 13; **e**: *n*_mCherry_ = 8, *n*_mCherry-RAB12_ = 12). Data in (**c**, **d**, **f**) and the right panel of (**e**) were analyzed using the Welch’s *t*-test, whereas data in (**a**, **b**) and the left panel of (**e**) were analyzed using the Mann–Whitney test. *p < 0.05; ***p < 0.001. Error bars represent the SEM.

To elucidate the cellular mechanism by which RAB12 regulates neurotransmission, we examined SV trafficking using a live-cell imaging assay. We employed vesicular glutamate transporter 1-pHluorin (vGLUT1-pHluorin)^19,20^ to monitor SV endocytosis, exocytosis, and recycling kinetics. vGLUT1 is an SV membrane protein responsible for glutamate loading, whereas pHluorin is a pH-sensitive GFP variant that fluoresces when SVs fuse with the presynaptic membrane (pH ~7.4) and that is quenched upon reacidification (pH <5.5)^19,21^. We expressed vGLUT1-pHluorin in WT and *Rab12*^−/−^ mouse primary cortical neurons and found no significant difference in the SV endocytosis time constant or the exocytosis fraction following stimulation with either 100 (10 Hz, 10 s) or 300 (10 Hz, 30 s) action potentials (APs) between WT and *Rab12*^−/−^ neurons (Fig. 3a, b). However, *Rab12*^−/−^ neurons exhibited a decreased SV exocytosis time constant, whereas the exocytosis fraction remained unchanged (Fig. 3c).

To further investigate the function of *Rab12* in SV trafficking, we overexpressed *Rab12* in primary cortical neurons. We observed no significant differences in the endocytosis time constant or in the exocytosis fraction between neurons overexpressing mCherry and neurons overexpressing mCherry-RAB12 (Fig. 3d–f). However, neurons overexpressing mCherry-RAB12 exhibited a significant increase in the exocytosis time constant compared to the mCherry control (Fig. 3f). Collectively, these results suggest that RAB12 inhibits SV exocytosis in neurons.

### Localization of RAB12 at the synaptic compartment

**Fig. 4:**
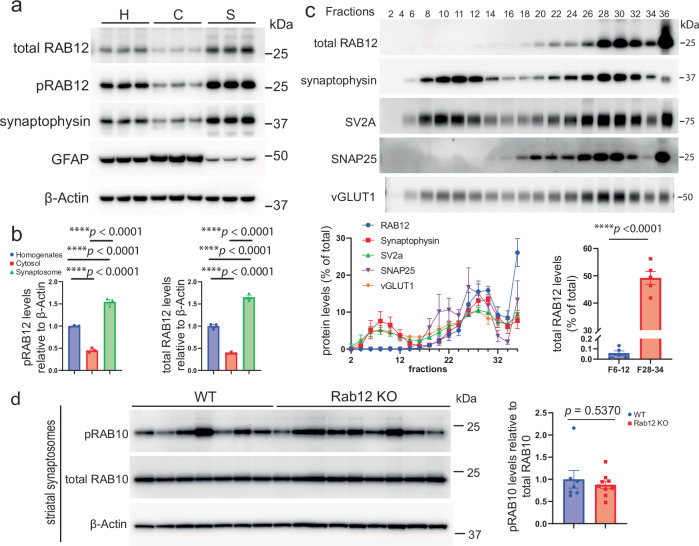
Subcellular localization of RAB12 in neurons. **a** Synaptosomes isolated from WT mouse cortex were fractionated, and levels of total RAB12, phosphorylated RAB12 (pRAB12), synaptophysin, and GFAP in homogenates (H), cytosol (C), and synaptosome (S) fractions were analyzed by Western blot (*n* = 3). **b** Quantification of relative protein levels. The band intensity of each respective protein in each sample was normalized to the corresponding β-Actin intensity and subsequently to the homogenate fraction. Data are expressed as fold changes relative to the homogenate fractions and presented as mean ± SEM. **c** SVs were isolated from mouse brains. Western blot was used to determine the levels and distribution of total RAB12 and synaptic proteins, including synaptophysin, SV2A, SNAP25, and vGLUT1, in every other isolated fraction. Equal volumes per fraction were loaded. Protein distribution is expressed as the percentage of total protein of interest across all fractions and is shown in the bottom left graph. The percentage of total RAB12 in fractions 6–12 and fractions 28–34 is shown in the bottom right graph (*n* = 5). **d** Western blot analysis of pRAB10 levels in striatal synaptosomes from WT and *Rab12* KO mice. Data in (**b**) were analyzed using the ordinary one-way ANOVA, whereas data in (**c**, **d**) were analyzed using the Welch’s t-test. ****p < 0.0001. Error bars represent the SEM.

A previous proteomics study of SVs isolated from rat brains suggested an association of RAB12 with SVs^22^. To seek additional evidence, we first isolated synaptosomes from mouse brains and performed immunoblot analysis. The results showed an enrichment of RAB12 and phosphorylated RAB12 (pRAB12) in the synaptosomes (Figs. 4a, b and S2a–c). Immunofluorescence (IF) imaging of primary neurons expressing mCherry-RAB12 showed a strong fluorescence signal in the soma and a discrete distribution in neurites, whereas the signal in neurons expressing control mCherry appeared mostly diffuse across all neuronal subcompartments. Moreover, neuritic mCherry-RAB12 partially colocalized with or was in close proximity to presynaptic markers such as synapsin and synaptophysin, as evidenced by a trend toward an increase in Pearson’s correlation coefficient compared to neuritic mCherry. We observed no difference in the average intensity of synapsin or synaptophysin between neurons expressing mCherry-RAB12 and those expressing mCherry alone (Fig. S2d, e).

To further examine the localization of RAB12 in neurons, we prepared SVs from mouse brains using an established protocol^23,24^. We separated free SVs from active zone-associated SVs using linear sucrose gradient ultracentrifugation. The analysis indicated a strong enrichment of RAB12 in fractions 28–34, similar to the distribution pattern observed for SNAP25, which represent docked SVs associated with the active zone or clustered SVs, whereas RAB12 was barely detectable in fractions 6–12, which represent free SVs (Fig. 4c). These data suggest that RAB12 is likely associated with the synaptic active zone in neurons.

Previous studies from our group and others demonstrated that RAB12 is an activator of LRRK2 activity^11,13,14^, and we found that pRAB10 levels (an LRRK2 substrate) are significantly reduced in *Rab12*^−/−^ astrocytes^11^. We next investigated whether RAB12 regulates LRRK2 activity toward phosphorylation of RAB10 in synaptic fractions in neurons. Unexpectedly, pRAB10 levels did not differ significantly between *Rab12*^−/−^ and WT mice in striatum lysates (Fig. S2f) or in striatal synaptosome fractions (Fig. 4d), suggesting that RAB12 regulation of LRRK2 activity may be cell-type specific.

### Impairment of synaptic protein homeostasis in the absence of RAB12

**Fig. 5:**
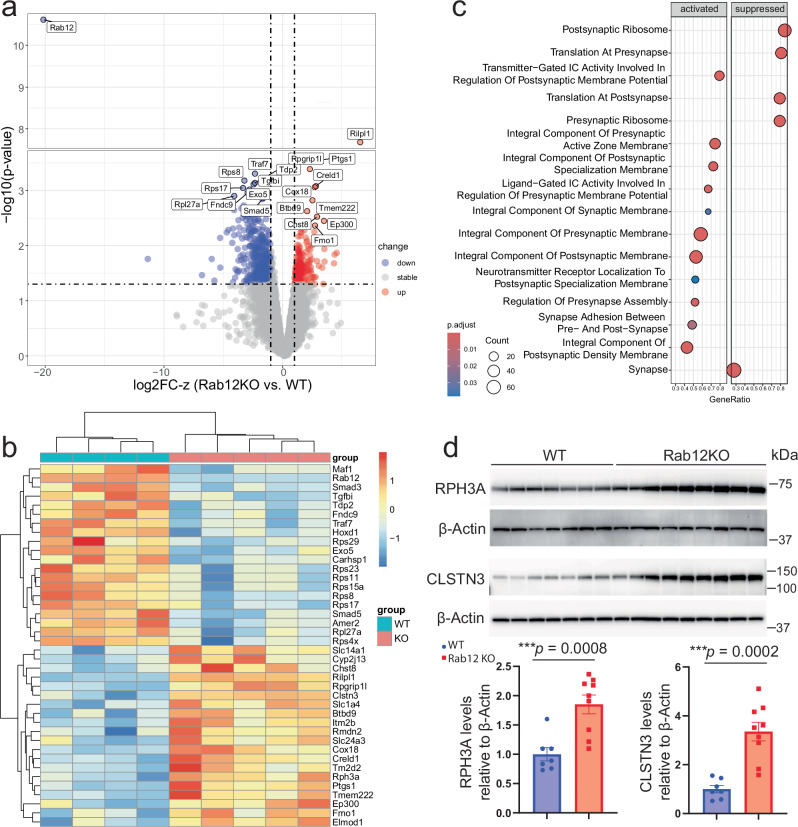
Proteomic analysis of striatal synaptosomes from *Rab12* KO mice. **a**, **b** Synaptosomes were isolated from striatal tissues of WT and *Rab12* KO mice for proteomic analysis. The volcano plot highlights the top 10 DEPs ranked by *p* value (**a**). The heatmap shows the top 20 upregulated and downregulated DEPs (**b**). Gene Set Enrichment Analysis (GSEA), using SynGO as the reference dataset, identified synaptic function-related pathways that were potentially activated (**c**, left) or suppressed (**c**, right). **d** Western blot analysis of RPH3A and CLSTN3 levels in striatal synaptosomes from WT and *Rab12* KO mice. Biological replicates: (1) proteomic analysis: *n*_WT_ = 4 (two males and two females), *n*_Rab12KO_ = 5 (two males and three females); (2) Western blot: *n*_WT_ = 7, *n*_Rab12KO_ = 9. DEPs in the proteomic analysis were defined using *p* < 0.05 and |z-score of log2FC| > 1 (**a**–**c**). In (**d**), data were analyzed using the Welch’s t-test. ***p < 0.001. Error bars represent the SEM.

We next conducted a proteomic analysis of *Rab12*^−/−^ brains to investigate the molecular basis underlying the role of *Rab12* in regulating synaptic trafficking. We isolated synaptosome fractions from the striatum of WT and *Rab12*^−/−^ mice and performed mass spectrometry. We identified numerous differentially expressed proteins (DEPs; *p* < 0.05; Fig. 5a, b, Data S1). Principal component analysis (PCA) revealed partial separation between WT (blue circles) and *Rab12*^−/−^ (red triangles) samples (Fig. S3a).

Gene Ontology (GO) analysis indicated that the upregulated DEPs were enriched in ion transmembrane transport (e.g., GO:0034765) and synaptic membrane (e.g., GO:0042734) (Fig. S3b; Data S2–S4). Using Gene Set Enrichment Analysis (GSEA)^25^ and SynGO^26^ as the reference dataset, we identified 16 entries associated with synaptic functions (Fig. 5c; Data S5). Eleven entries were categorized as activated (Fig. 5c, left) and five as suppressed (Fig. 5c, right), suggesting a potential enhancement of synapse function. These activated entries were primarily associated with integral components and assembly of presynaptic and postsynaptic membranes.

We next performed immunoblot analysis to validate potential changes in candidate synaptic proteins in *Rab12*^−/−^ synaptosomes. Both Rabphilin-3A (RPH3A) and Calsyntenin-3 (CLSTN3) were shown to be upregulated in the proteomics analysis (Fig. 5b, Data S1). They were significantly elevated in *Rab12*^−/−^ synaptosomes by Western blot analysis (Fig. 5d). RPH3A is a RAB3A effector predominantly localized at presynaptic terminals^27^ and known to regulate neurotransmitter release^28,29^. CLSTN3 is a postsynaptic adhesion molecule that promotes excitatory synapse formation in the hippocampus^30^. Rab Interacting Lysosomal Protein Like 1 (RILPL1), a known effector of RAB12^31^, was also upregulated in the proteomic dataset in *Rab12*^−/−^ synaptosomes (Fig. 5a, b; Data S1). However, Western blot analysis revealed no change in RILPL1 levels (Fig. S3d).

## Discussion

RAB12 is a physiological substrate of the LRRK2 kinase, and RAB12 may mediate the LRRK2 pathogenic pathway in glia and peripheral tissues^11,32^. Yet the function of RAB12 in neurons has not been characterized. Here, we demonstrate that RAB12 negatively regulates neurotransmission and that loss of RAB12 induces locomotor hyperactivity in mice. Mechanistically, RAB12 regulates presynaptic glutamate release, likely through inhibiting SV exocytosis. RAB12 is associated with SVs and is particularly enriched in synaptic active zone fractions. Moreover, we show that RAB12 regulates the homeostasis of specific presynaptic proteins, underlying its function in regulating SV exocytosis.

Our observation of RAB12 in regulating SV exocytosis is consistent with an early report that RAB12 promotes microtubule-dependent retrograde vesicle transport and inhibits the exocytosis of secretory granules in mast cells upon immune stimulation^6^. The proteomics analysis of synaptosomes lacking *Rab12* revealed alterations in synaptic pathways, providing a potential mechanism underlying this effect. We validated the increase in RPH3A and CLSTN3 proteins in the striatal lysates from *Rab12* KO mice. RPH3A localizes to SVs^27^; it promotes vesicle docking and fusion with the plasma membrane^28^, regulates SV release by interacting with RAB3A^29^, and interacts with the soluble N-ethylmaleimide-sensitive factor attachment protein receptor (SNARE) complex^33,34^. CLSTN3, a postsynaptic adhesion molecule, promotes presynaptic differentiation, contributes to the development and balance of excitatory and inhibitory synapses^35^, and enhances excitatory synapse formation in hippocampal neural circuits^30^. Therefore, it is plausible that the increase in RPH3A and/or CLSTN3 could contribute to the enhanced synaptic transmission phenotypes observed in *Rab12* KO mice. However, further studies are needed to validate this hypothesis.

Although our finding reveals a role of RAB12 in regulating synaptic trafficking and enhancing synaptic transmission, our study has several limitations. First, since *Rab12* is globally knocked out in our mouse model, we cannot determine the cell type, neuron type, or neural circuitry that contributes to the locomotor hyperactivity in *Rab12* KO mice. The employment of cell-type-specific *Rab12* KO models will be required to delineate the contributions of different cell types to this behavioral phenotype in future studies. Second, while our electrophysiological and imaging data imply an increased presynaptic release probability, it is also possible that *Rab12* KO alters SV abundance at presynaptic terminals, thereby facilitating synaptic transmission. Quantification of SVs at synapses using electron microscopy will be required to address this question. Finally, our study has yet to determine whether this function is mechanistically linked to LRRK2, given prior reports that LRRK2 regulates synaptic activity^3–5,36^. For example, whether LRRK2 phosphorylation of RAB12 modulates RAB12 function in SV trafficking is unclear. Furthermore, our current study showed that in contrast to astrocytes^11^, synaptosomes isolated from *Rab12* KO striatal neurons showed no change in phosphorylated RAB10 levels, suggesting cell type-dependent regulation of LRRK2 activity by RAB12. Therefore, it also remains to be tested whether RAB12 could mediate pathological LRRK2 function in deregulating synaptic transmission^3–5^. Future experiments are warranted to address these questions and provide deeper insight into the pathogenic mechanisms of LRRK2-linked PD.

## Methods

### Animals

Mice were housed in a temperature- (20–23 °C), humidity- (40–60%), and light-controlled (12 h light/12 h dark cycle) environment with food and water available ad libitum. All animal procedures were conducted in accordance with protocols approved by the Institutional Animal Care and Use Committees (IACUC) of the Icahn School of Medicine at Mount Sinai (IACUC-2021-00031), Thomas Jefferson University (IACUC-2021-01452), and the University of Georgia (IACUC-A2023-05-031-Y1-A1). All efforts were made to minimize animal use and reduce potential pain or distress. *Rab12* KO mice were generated by our group^11^, and their WT littermates served as controls.

### Behavioral tests

Male and female *Rab12* KO mice and their WT littermates at 3 and 6 months of age underwent behavioral assessments to evaluate general locomotion by the open field test, general development by body weight measurement, motor coordination by the rotarod test, and muscle strength by the grip strength test. (1) Open field test. As previously described^5^, mice were habituated in a quiet, dark room illuminated with red lighting for 1 h to acclimate to the testing environment. Then, each mouse was placed in a 16 × 16 inch transparent chamber monitored by a Versamax system (Accuscan). The mouse’s movements were recorded for 1 h. (2) Body weight measurement. Mice were placed on a digital scale to record their body weight. (3) Rotarod test. As previously described^5^, mice were placed individually on the rod of the rotarod device. Each rod slot contained one mouse. After the examination program started, the rod accelerated from 4 to 40 rpm over 5 min. The time each mouse remained on the accelerating rod was recorded. Each mouse underwent three trials spaced 20 min apart, and the results were averaged. All mice underwent three training trials before the trials for data collection. (4) Grip strength test. Mice were placed on the grip bar of a grip strength device (BIOSEB, Model GT3) and allowed to grasp the grip with their forepaws. Then, the mice were gently pulled backward horizontally until they released their grip. Each mouse underwent five consecutive trials. The average grip strength per mouse was used for analysis.

### Slice preparation

Male and female wild-type (WT) and *Rab12* KO mice (4–10 months old) were anesthetized with ketamine (100 mg/kg) and xylazine (10 mg/kg), and transcranially perfused with ice-cold, oxygenated cutting solution^37,38^. Coronal brain slices (300 µm thick) were prepared using a vibratome (VT1200, Leica, Solms, Germany) and recovered in oxygenated artificial cerebrospinal fluid (ACSF) at 36 °C for 30 min. All solutions were continuously bubbled with 95% O₂/5% CO₂ to maintain oxygenation and pH.

### Whole-cell patch-clamp recordings

Whole-cell patch-clamp recordings were performed on medium spiny neurons (MSNs) in the dorsolateral striatum of acute brain slices using an upright microscope (BX50WI, Olympus, Japan) equipped with infrared differential interference contrast optics and a 40× water immersion objective. Recordings were conducted at 35–36 °C in a submerged chamber continuously perfused with ACSF (1–2 mL/min).

All recordings were made using a Multiclamp 700B amplifier (Molecular Devices, Foster City, CA) and digitized at 10 kHz with a Digidata 1440 A (Molecular Devices). Data were acquired using Clampex 10.2 software (Molecular Devices) for subsequent analysis.

Current-clamp recordings were used to assess intrinsic membrane properties. Pipettes (3–5 MΩ) were filled with an internal solution containing (in mM): 115 K-gluconate, 20 KCl, 10 HEPES, 2 MgCl₂, 2 Mg-ATP, 1 Na₂-ATP, and 0.3 GTP (pH 7.3, 280–290 mOsm). Input resistance was calculated from responses to 100 pA, 100 ms hyperpolarizing pulses. Rheobase was determined using a ramp protocol, and the number of evoked spikes was measured following depolarizing current steps. Recordings were excluded if access resistance changed by >20% during the experiment. Bridge balance was not compensated.

Voltage-clamp recordings were used to assess excitatory synaptic activity. sEPSCs were recorded at –80 mV in the presence of bicuculline (50 μM) or SR-95531 (10 μM) to block GABA_A receptor-mediated transmission. mEPSCs were isolated by adding tetrodotoxin (TTX, 0.5–1 µM) to block action potentials. For mEPSC recordings, pipettes were filled with a cesium-based internal solution containing (in mM): 120 CsMeSO₃, 5 NaCl, 10 HEPES, 1.1 EGTA, 2 Mg-ATP, 0.3 Na-GTP, 2 Na-ATP, and 5 QX-314 (pH 7.3, 280–285 mOsm). Recordings were run with gap-free protocol. Miniature events were detected and analyzed using Mini Analysis program (Synaptosoft, Decatur, GA). The threshold for amplitude detection of an event was generally adjusted to twice the RMS noise level (typically 8 pA). All data shown, except mEPSCs and sEPSCs, were analyzed with Clampfit 10.2 software (Molecular Devices). Signals were filtered at 3 kHz and digitized at 20 kHz.

To assess synaptic release probability, evoked EPSCs were recorded from MSNs in response to electrical stimulation of the corpus callosum using a bipolar platinum electrode. Paired-pulse stimulation protocols were applied at interstimulus intervals (ISIs) of 20, 50, 100, 150, and 200 ms. Paired-pulse ratios (EPSC2/EPSC1) were calculated to infer presynaptic release probability.

Pharmacological agents, including TTX, bicuculline, SR-95531, were purchased from Tocris Bioscience (Bristol, UK).

### Cloning and constructs

AAV-hSyn-mCherry was obtained from Addgene (#114472). FUGW-vGLUT1-pHluorin was a gift from Dr. Robert Edwards (UCSF)^20^. DNA fragments encoding GST, human Rab12, mCherry, IRES, and tTA were amplified using PCR with Q5® High-Fidelity DNA Polymerase (NEB, #M0491S). The DNA fragments of mCherry-GST-IRES-tTA and mCherry-Rab12-IRES-tTA were inserted into a linearized AAV plasmid backbone derived from AAV-hSyn-IRES-mCherry (a gift from Dr. Lingchun Kong in Dr. Lan Maze’s lab at the Icahn School of Medicine at Mount Sinai). This plasmid was linearized by digestion with NheI and EcoRI restriction enzymes, removing the IRES-mCherry sequence. Constructs were assembled using the NEBuilder® HiFi DNA Assembly Kit (NEB, #E2621L) to generate AAV-hSyn-mCherry-GST-IRES-tTA and AAV-hSyn-mCherry-Rab12-IRES-tTA. To generate AAV-TRE-vGLUT1-pHluorin, DNA fragments encoding fDIO-GFP-IRES-tTA were excised from the AAV-TRE-fDIO-GFP-IRES-tTA plasmid (Addgene, #118026) using EcoRI and EcoRV restriction enzymes. vGLUT1-pHluorin DNA fragments were amplified and inserted into the linearized plasmid using the NEBuilder® HiFi DNA Assembly Kit.

### Virus packaging

Lentivirus and AAV production were conducted as previously described^11^. HEK-293T cells (ATCC, #CRL-11268) were maintained in DMEM (GIBCO, #11965-092) supplemented with 10% fetal bovine serum (FBS, ATLANTA, #S11550) and used for viral packaging upon reaching 90% confluence. For lentiviral packaging of FUGW-vGLUT1-pHluorin, the procedure was performed as previously described^11^. FUGW-vGLUT1-pHluorin, pCAG-VSVG (Addgene, #64084), pRSV-Rev (Addgene, #12253), and pMDLg/pRRE (Addgene, #12251) were co-transfected into HEK-293T cells using Lipofectamine 3000 (Thermo Fisher Scientific, #L3000008). Six hours post-transfection, the medium was replaced with fresh culture medium. Viral supernatants were collected at 24, 48, and 60 h post-transfection, combined, and centrifuged at 1000 × *g* for 10 min at 4 °C. The supernatant was ultracentrifuged at 75,600 × *g* for 2 h at 4 °C. The supernatant was then discarded, and the lentiviral pellet was resuspended in lenti-freezing medium (0.5 M sucrose in DMEM), aliquoted, snap-frozen in liquid nitrogen, and stored at −80 °C.

AAV packaging was performed as previously described^11^. Briefly, AAV-hSyn-mCherry, AAV-hSyn-mCherry-GST-IRES-tTA, AAV-hSyn-mCherry-Rab12-IRES-tTA, or AAV-TRE-vGLUT1-pHluorin were co-transfected with pAAV2/1 (Addgene, #112862) and pAdDeltaF6 (Addgene, #112867) into HEK-293T cells. The culture medium was then replaced with fresh medium at 6 h after transfection. Forty-eight hours after transfection, viral supernatants were collected. Cell debris was pelleted by centrifugation at 1000 × *g* for 10 min at 4 °C, and the supernatant was transferred to a new tube. AAV particles were precipitated overnight at 4 °C using polyethylene glycol 8000 (PEG8000, Sigma, #89510)-NaCl buffer (40% PEG8000, 2.5 M NaCl). The precipitated AAV particles were pelleted by centrifugation at 4000 × *g* for 1 h at 4 °C. Following removal of the supernatant, AAV particles were resuspended in neuronal culture medium consisting of MEM (Gibco, #11090081), glucose (5 mg/mL), 1 × GlutaMAX supplement (Gibco, 35050-061), 1 × B-27 Supplement (Gibco, 17504044), transferrin (0.1 mg/mL, Calbiochem, #616420), insulin (24 μg/mL, Sigma, #16634), and 10% heat-inactivated FBS (ATLANTA, #S11510H). Virus aliquots were frozen and stored at −80 °C.

### Primary cortical neuron culture and infection

Primary cortical neurons were cultured as previously described^39,40^. Cortical tissues were dissected from postnatal day 0 (P0) neonatal mice of either sex and digested using trypsin (Sigma, #T1005) and deoxyribonuclease I (DNase I, Sigma, #D5025). Cells were seeded at a density of 142,000 cells/cm^2^ and maintained in neuronal culture medium. Virus infection was performed on day in vitro (DIV) 5. For pHluorin assays comparing WT and *Rab12* KO neurons, cortical neurons were infected with FUGW-vGlut1-pHluorin. For pHluorin assays comparing mCherry and mCherry-RAB12 overexpression, neurons were co-infected with AAV-TRE-vGLUT1-pHluorin and either AAV-hSyn-mCherry-GST-IRES-tTA or AAV-hSyn-mCherry-RAB12-IRES-tTA to ensure co-expression of vGLUT1-pHluorin and mCherry fusion proteins in individual neurons via the tTA-mediated gene expression system. The amount of each virus used to treat neurons was equal between groups within the same cohort to minimize differences in pHluorin expression levels between groups. On DIV 6, the culture medium was replaced with neuronal culture medium supplemented with 5% FBS and 4 μM cytosine β-D-arabinofuranoside hydrochloride (Ara-C, Sigma-Aldrich, #C6645). Neurons were used for live-cell imaging on DIV 10-11.

### Live cell imaging

Live-cell imaging was performed as previously described^5,39^. Neurons were placed in a custom-built laminar chamber, allowing continuous buffer perfusion, and maintained in Tyrode’s salt solution consisting of 119 mM NaCl (Sigma, #S7653), 2.5 mM KCl (Sigma, #P9333), 2 mM CaCl_2_ (Sigma, #C3881), 2 mM MgCl_2_ (Sigma, #M2670), 25 mM HEPES (Sigma, #H7006), 30 mM D-glucose (Sigma, #G5400), 10 μM CNQX disodium salt hydrate (Sigma, #C239) and 50 μM DL-2-Amino-5-phosphonopentanoic acid (Sigma, #A5282), with the pH adjusted to 7.40. The chamber temperature was maintained at 28 °C. Field stimulation was applied using an A310 Accupulser and an A385 stimulus isolator (World Precision Instruments). Live-cell imaging was conducted using a highly sensitive, back-illuminated EM-CCD camera (iXon model #DU-897E-BV, Andor) installed on an IX73 microscope with laser illumination (Olympus) and a laser combiner system (Andor). Endocytosis was assessed using 100 action potentials (APs) at 10 Hz for 10 s and 300 APs at 10 Hz for 30 s, whereas exocytosis was evaluated using 1200 APs at 10 Hz for 120 s in the presence of 1 μM bafilomycin. The duration of each stimulation pulse was 1 ms, and image acquisition was controlled using iQCOR E-FST iQ3.0 software (version 2.x, Andor), with images acquired at 500 ms per frame. After each stimulation protocol, NH_4_Cl buffer was perfused into the chamber to determine the total pool of vGLUT-pHluorin-expressing vesicles for normalization of data from the 100 AP, 300 AP, and 1200 AP trials. Acquisition settings were kept consistent across groups.

### Synaptosome fractionation

Synaptosome fractionation was performed on cortical or striatal tissues from WT mice using Syn-PER™ Synaptic Protein Extraction Reagent (Thermo Fisher Scientific, #87793). Tissues were homogenized in chilled Syn-PER Reagent (20 μL/mg tissue) supplemented with Halt™ protease and phosphatase inhibitor cocktail (Thermo Fisher Scientific, #78440) using a Dounce homogenizer. The homogenate was centrifuged at 1200 × *g* for 10 min at 4 °C. An aliquot of the supernatant was saved as the homogenate fraction, whereas the remaining supernatant was centrifuged at 15,000 × *g* for 20 min at 4 °C. The resulting supernatant, representing the cytosol fraction, was transferred to a new tube, while the pellet constituted the synaptosome fraction. For the samples intended for proteomics analysis, synaptosome pellets were snap-frozen and stored at −80 °C until further processing. For the samples intended for Western blotting, synaptosome pellets were resuspended in Syn-PER Reagent for protein concentration assessment.

### Subcellular fractionation of synaptic vesicles

Isolation of synaptic vesicles was performed as previously described^23^. One mouse brain of 3-month-old WT mice (strain C57BL/6J; JAX) was homogenized in preparation buffer (5 mM Tris-HCl, pH 7.4, 320 mM sucrose). The homogenate was centrifuged for 10 min at 3600 g_av_. The supernatant was collected, and the pellet was resuspended in preparation buffer and recentrifuged. Both supernatants were pooled, and the final pellet was discarded. Discontinuous Percoll gradients were prepared by layering 7.5 ml supernatant onto three layers of 7.5 ml Percoll solution (3, 10, and 23% v/v in 320 mM sucrose, 5 mM Tris-HCl, pH 7.4). After centrifugation for 7 min at 31,400 g_av_, cloudy fractions containing synaptosomes were collected, diluted in four volumes of preparation buffer, and centrifuged for 35 min at 20,000 g_av_. Synaptosomal pellets were subjected to osmotic lysis by resuspending pellets in 60 ml of lysis buffer (5 mM Tris-HCl, pH 7.4) and pipetting 30× up and down. The suspension was centrifuged for 1 h at 188,000 g_av_, and the pellet was resuspended in 4 ml of sucrose gradient buffer (200 mM sucrose, 0.1 mM MgCl_2_, 0.5 mM EGTA, 10 mM HEPES-NaOH, pH 7.4) and homogenized. The resulting crude vesicle suspension was layered onto a continuous sucrose gradient ranging from 0.3 M to 1.2 M sucrose (sucrose in 0.5 mM EGTA, 10 mM HEPES-NaOH, pH 7.4) and centrifuged for 2 h at 85,000 g_av_. Thirty-six fractions (1 ml each) were collected from top to bottom of the gradient. Every second fraction of the gradient was analyzed by quantitative immunoblotting.

### Western blotting

Protein concentrations of the samples were determined using the Pierce™ BCA Protein Assay Kit (Thermo Fisher Scientific, #23227) and adjusted to equal concentrations with ddH_2_O. 3 × LDS sample buffer prepared by mixing 750 μL 4 × NuPAGE™ LDS Sample Buffer (Thermo Fisher Scientific, #NP0007), 150 μL 1 M dithiothreitol (DTT, Sigma, #11583786001), and 100 μL ddH2O was added to each sample to achieve a final concentration of 1 ×. Samples were incubated at 70 °C for 10 min, then centrifuged and briefly vortexed. Equal amounts of total protein from each sample were loaded onto a NuPAGE gel, separated by SDS-PAGE, and transferred onto PVDF membranes. Membranes were blocked with Intercept^®^ Blocking Buffer (LI-COR, #927-60001) at room temperature for 1 h, followed by overnight incubation at 4 °C with primary antibodies: rabbit anti-pRAB12 (Abcam, #ab256487, 1:1000), mouse anti-RAB12 (Santa Cruz Biotechnology, #sc-515613, 1:1000), mouse anti-β-Actin (Cell Signaling Technology, #3700S, 1:10,000), mouse anti-synaptophysin (Synaptic Systems, #101011, 1:1000), rat anti-GFAP (Invitrogen, #13-0300, 1:1000), rabbit anti-RILPL1 (BOSTER, #A13355, 1:1000), rabbit anti-β-Actin (Cell Signaling Technology, #4970S, 1:10,000), mouse anti-PSD95 (Millipore, #MAB1598, 1: 1000), rabbit anti-SV2A (SYSY, #119002, 1: 3000), Guinea pig anti-vGLUT1(SYSY, #135304, 1:1000), rabbit anti-CLSTN3 (Proteintech, #13302-1-AP, 1:800), rabbit anti-RPH3A (Proteintech, #11396-1-AP, 1:2000), rabbit anti-synaptotagmin-1 (CST, #3347S), and rabbit anti-SNAP25 (SYSY, #111002, 1:5000). Following primary antibody incubation, the membranes were washed three times with TBST, 5 min each, and incubated with appropriate HRP-conjugated secondary antibodies (Cell Signaling Technology, #7074S for pRAB12, RILPL1, SV2A, CLSTN3, RPH3A, synaptotagmin-1, and SNAP25; #7076S for total RAB12, synaptophysin, PSD95, and mouse anti-β-Actin; Santa Cruz Biotechnology, #sc-2006 for GFAP) for 1 h at room temperature. Membranes were washed three times with TBST for 5 min each. Signals were detected using SuperSignal™ West Pico Chemiluminescent Substrate (Thermo Fisher Scientific, #34580).

### Immunofluorescent staining

Primary neurons were washed twice with chilled PBS containing 4% sucrose, 0.5 mM CaCl_2_, and 1 mM MgCl_2_, then fixed with 4% paraformaldehyde and 4% sucrose in PBS for 10 min at room temperature. After three washes with PBS for 5 min each, cells were blocked for 1 h at room temperature in blocking buffer [5% bovine serum albumin (BSA, Sigma, #A9647) and 0.1% Triton X-100 in PBS]. After removing the blocking buffer, cells were incubated overnight at 4 °C with primary antibodies: guinea pig anti-synapsin1/2 (Synaptic Systems, #106004, 1:1000), and goat anti-mCherry (SICGEN, #AB0040-200, 1:1000). Cells were washed three times with PBST (PBS containing 0.05% Triton X-100) for 3 min each and incubated with Alexa-fluorescein-conjugated secondary antibodies (Thermo Fisher Scientific, #A11017 for synaptophysin, #A11073 for synapsin1/2, and #A11058 for mCherry) for 1 h at room temperature. Excess secondary antibodies were washed off by three PBST washes for 3 min each. Nuclei were counterstained with 10 µM Hoechst solution (Thermo Fisher Scientific, #62249) for 5 min at room temperature. Cells were mounted using ProLong™ Diamond Antifade Mountant (Thermo Fisher Scientific, #P36961).

### Image acquisition and analysis

Western blot images were captured using the ChemiDoc Imaging system (Bio-Rad). Immunofluorescent images were acquired using a Zeiss LSM 780 confocal microscope (Carl Zeiss) with a 63× objective at a resolution of 1024 × 1024 in z-stack mode. Western blot band intensities were quantified using Fiji (ImageJ; https://imagej.nih.gov/ij/index.html). Specifically, in the synaptosome fractionation and proteomic validation experiments, the band intensity of each target protein was normalized to the corresponding β-actin band to obtain a normalized ratio. Relative protein levels were then calculated by dividing the normalized ratio by the mean normalized ratio of the control group. For the SV isolation, the intensity of each target protein band was divided by the total band intensity across all fractions and multiplied by 100 to determine the percentage in each fraction. The fluorescence intensities of the presynaptic markers synaptophysin and synapsin were measured using Fiji’s particle analysis plugin, whereas Pearson’s correlation coefficient for protein colocalization was calculated using the JaCoP plugin^41^.

### Whole-proteome profiling using 18-plex TMTpro coupled with LC/LC-MS/MS

Four WT (two males and two females) and five *Rab12* KO mice (two males and three females) aged 3 months were included in the proteomic analysis. All samples were obtained from the striatum and were processed under identical experimental conditions in a single batch, including tissue collection, synaptosome fractionation, and proteomic analysis. Whole-proteome profiling was performed as described previously^42,43^. Fresh lysis buffer (8 M urea, 50 mM HEPES, 0.5% sodium deoxycholate, 1× phosphatase inhibitor cocktail, pH 8.5) using for lysate striatal synaptosome (*n* = 5 for *Rab12* KO, *n* = 4 for WT). 100 µL of lysis buffer was added for the striatal synaptosome pellets from each mouse. Glass beads were included to facilitate tissue homogenization using a Bullet Blender in this process. Protein concentrations were determined by BCA assay (Thermo Fisher Scientific), and 50 µg of protein from each sample was loaded onto a 10% Bis-Tris gel (BIO-RAD) and electrophoresed for 10 min to obtain short gel bands. Gel bands of each sample were cut and clean-up individually, followed by reduction and alkylation using DTT and iodoacetamide (IAA), respectively. Proteins were digested in-gel using trypsin at a 50:1 ratio (protein: enzyme, w:w) overnight. The supernatant from in-gel digestion was dried and resuspended in HEPES buffer (pH 8.5) for 18-plex TMTpro labeling peptides at ratio of 1:1.5 (w:w, protein: TMT reagent) for 30 min at room temperature. After TMT labeling, the samples were mixed equally, desalted using a Sep- Pak C18 cartridge (50 mg, Waters), and dried. The pooled sample was separated on a BEH C18 column (3 × 150 mm, 1.7 µm, part NO.186004690) in 125 min using a gradient from 5% to 65% Buffer B at flow rate of 0.15 µL/min (Buffer A: 10 mM NH_4_COOH, pH 8.0; Buffer B: 10 mM NH_4_COOH, pH 8.0, 90%ACN), and 40 fractions were collected. Each fraction was then eluted under acidic conditions at 65 °C in 95 min with a gradient from 8 to 65% Buffer B (Buffer A:0.2% formic acid, 3% DMSO; Buffer B: 67% ACN, 0.2% formic acid, 3% DMSO) at a flow rate of 0.25 µL/min using a C18 column (75 µm ID* 15 cm, 1.7 µm, CoAnn) coupled to a nanoRSLC-Q-Exactive-HF system. Mass spectrometry (MS) data were acquired in positive ion mode with a resolution of 60,000 at a scan range of 460–1600 m/z for MS1 (AGC target: 1.0E6). The top 20 MS1 precursors were selected for MS2 (scan range: 200–2000 m/z; isolation window: 1.0 m/z; isolation offset: 0.2 m/z), with a maximum ion injection time of 110 ms and a dynamic exclusion of 10 s. Normalized collision energy (NCE) was set to 32.

### Proteomic data analysis

MS data search, filtering, and quantification were performed using the JUMP suite^44^. For the database search, dynamic methionine oxidation (+15.99492 Da), static TMTpro modifications on peptide N-termini and lysine residues (+304.20715 Da), and cysteine alkylation (+57.02146 Da) were specified. For filtering, an initial false discovery rate (FDR) of 0.2 was applied, followed by multistep FDR filtering and fully removal of one-hit wonders. A minimum peptide length of six amino acids was required, allowing up to two missed cleavages and a Jscore threshold >10. For quantification, precursor peak intensity threshold was set at 50%, with minimum and median intensities of peptide-spectrum matches (PSMs) at 1000 and 5000, respectively, and minimum and median protein intensities at 2000 and 10,000.

All downstream analyses were performed in R (version 4.5.0 and RStudio https://posit.co/download/rstudio-desktop/). Proteins with *p* < 0.05 and |z-score of log2FC| > 1 were considered statistically significant. Principal component analysis was performed using the draw_pca function from the tinyarray package (https://github.com/xjsun1221/tinyarray). The volcano plot was generated using the ggplot2 package, highlighting the top 10 upregulated and top 10 downregulated DEPs. The top 20 upregulated and top 20 downregulated DEPs, ranked by *p* values, were visualized in a heatmap using the pheatmap package (DOI: 10.32614/CRAN.package.pheatmap). Gene Ontology (GO) and Gene Set Enrichment Analysis (GSEA) were performed using the clusterProfiler package^45^, with SynGO from Enrichr^26^ used as the referral gene set.

### Statistical analysis

Data are presented as mean ± SEM and/or as box plots, as indicated. Statistical comparisons between two groups were performed using the Welch’s *t*-test when data were normally distributed. Otherwise, the Mann–Whitney U test was used. Comparisons among three groups were performed using the ordinary one-way ANOVA when data exhibited normal distribution and homogeneity of variance. The Welch’s one-way ANOVA was used when data exhibited normal distribution but lacked homogeneity of variance. The Kruskal–Wallis test was used when data did not exhibit normal distribution. Dunnett’s or Tukey’s multiple comparisons test was used as post hoc tests following the ANOVA, where appropriate, whereas Dunn’s multiple comparisons test was used following the Kruskal–Wallis test. Two-way ANOVA was used for repeated-measures analyses. A significance threshold was set at *p* < 0.05. For electrophysiological experiments, unless otherwise specified, each condition included recordings from at least six neurons or slices obtained from a minimum of three independent animals. The specific statistical tests used for each dataset are described in the respective figure legends.

## Supplementary information

Supplementary Information

Data S1

Data S2

Data S3

Data S4

Data S5

Data S6

Data S7

Data S8

## Data Availability

The mass spectrometry proteomics data have been deposited to the ProteomeXchange Consortium via the PRIDE partner repository with the dataset identifier PXD075183. All other data of this study are indicated in the article and its Supplementary Information and supplementary files.

## Code availability

JUMPSuite (https://github.com/JUMPSuite/JUMP) was applied to analyze the proteomic data.
