# Supplementary material for: Disruption of the LRRK2 substrate RAB12 facilitates neurotransmission and causes hyperactivity in mice
Source: NPJ Parkinsons Dis. 2026 Apr 24;12:159. doi: 10.1038/s41531-026-01353-4 (PMC13319756; doi:10.1038/s41531-026-01353-4)
Supplement: Supplementary file 1 — Supplementary Information [file 41531_2026_1353_MOESM1_ESM.pdf]

Supplementary Information

Title: Disruption of the LRRK2 substrate RAB12 Facilitates Neurotransmission and Causes Hyperactivity in Mice

Li et al.

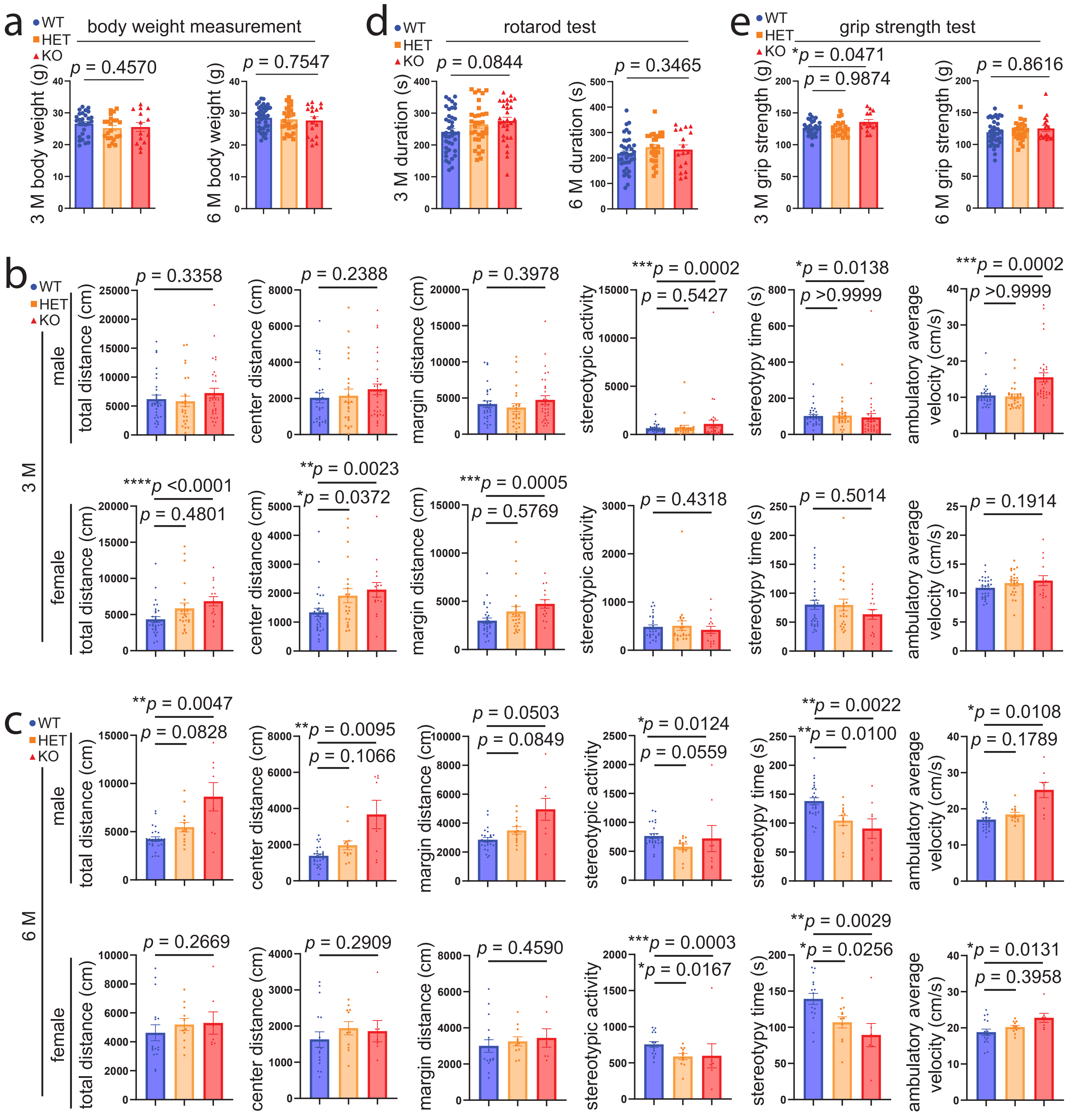

a
body weight measurement
WT
HET
KO
p = 0.4570
3 M body weight (g)
p = 0.7547
6 M body weight (g)
d
rotarod test
p = 0.0844
3 M duration (s)
p = 0.3465
6 M duration (s)
e
grip strength test
*p = 0.0471
p = 0.9874
3 M grip strength (g)
p = 0.8616
6 M grip strength (g)
b
3 M
male
female
p = 0.3358
total distance (cm)
p = 0.2388
center distance (cm)
p = 0.3978
margin distance (cm)
***p = 0.0002
p = 0.5427
stereotypic activity
*p = 0.0138
p >0.9999
stereotypy time (s)
***p = 0.0002
p >0.9999
ambulatory average velocity (cm/s)
****p <0.0001
p = 0.4801
**p = 0.0023
*p = 0.0372
***p = 0.0005
p = 0.5769
p = 0.4318
p = 0.5014
p = 0.1914
c
6 M
**p = 0.0047
p = 0.0828
**p = 0.0095
p = 0.1066
p = 0.0503
p = 0.0849
*p = 0.0124
p = 0.0559
**p = 0.0022
**p = 0.0100
*p = 0.0108
p = 0.1789
p = 0.2669
p = 0.2909
p = 0.4590
***p = 0.0003
*p = 0.0167
**p = 0.0029
*p = 0.0256
*p = 0.0131
p = 0.3958

Fig. S1 | Body weight, rotarod, grip strength, and sex-stratified open-field analysis of *Rab12* KO mice. a Body weight measurement of WT, *Rab12*$^{+/-}$, and *Rab12*$^{-/-}$ mice. At 3 months: $n_{WT} = 28$, $n_{HET} = 22$, $n_{KO} = 14$; at 6 months: $n_{WT} = 38$, $n_{HET} = 27$, $n_{KO} = 17$. **b**-**c** Sex-stratified analysis of the open-field data from Fig. 1. **b** At 3 months: males, $n_{WT} = 30$, $n_{HET} = 25$, $n_{KO} = 32$; females, $n_{WT} = 30$, $n_{HET} = 22$, $n_{KO} = 15$. **c** At 6 months: males, $n_{WT} = 26$, $n_{HET} = 13$, $n_{KO} = 8$; females, $n_{WT} = 16$, $n_{HET} = 11$, $n_{KO} = 7$. **d**-**e** Rotarod (**d**) and grip strength (**e**) tests of WT, *Rab12*$^{+/-}$, and *Rab12*$^{-/-}$ mice. Rotarod test: at 3 months, $n_{WT} = 40$, $n_{HET} = 32$, $n_{KO} = 29$; at 6 months, $n_{WT} = 38$, $n_{HET} = 27$, $n_{KO} = 17$. Grip strength test: at 3 months, $n_{WT} = 28$, $n_{HET} = 22$, $n_{KO} = 14$; at 6 months, $n_{WT} = 39$, $n_{HET} = 27$, $n_{KO} = 17$. Each data point represents one mouse. Statistical analyses were conducted after outlier removal (ROUT, Q = 1%), while outliers were retained in the graphs. The Welch's ANOVA was used for the left panel of (**a**) and for margin distance and ambulatory velocity in the upper panel of (**c**). The ordinary one-way ANOVA was used for the right panel of (**a**), total distance and ambulatory velocity in the lower panel of (**b**), stereotypic activity and ambulatory velocity in the lower panels of (**c**), stereotypic time in the upper and lower panels of (**c**), panel (**d**), and the left panel of (**e**). The Kruskal-Wallis test was used for the remaining panels in (**a**-**e**). *p* values are indicated in the corresponding graphs. *$p < 0.05$; **$p < 0.01$; ***$p < 0.001$; ****$p < 0.0001$. Error bars represent the SEM.

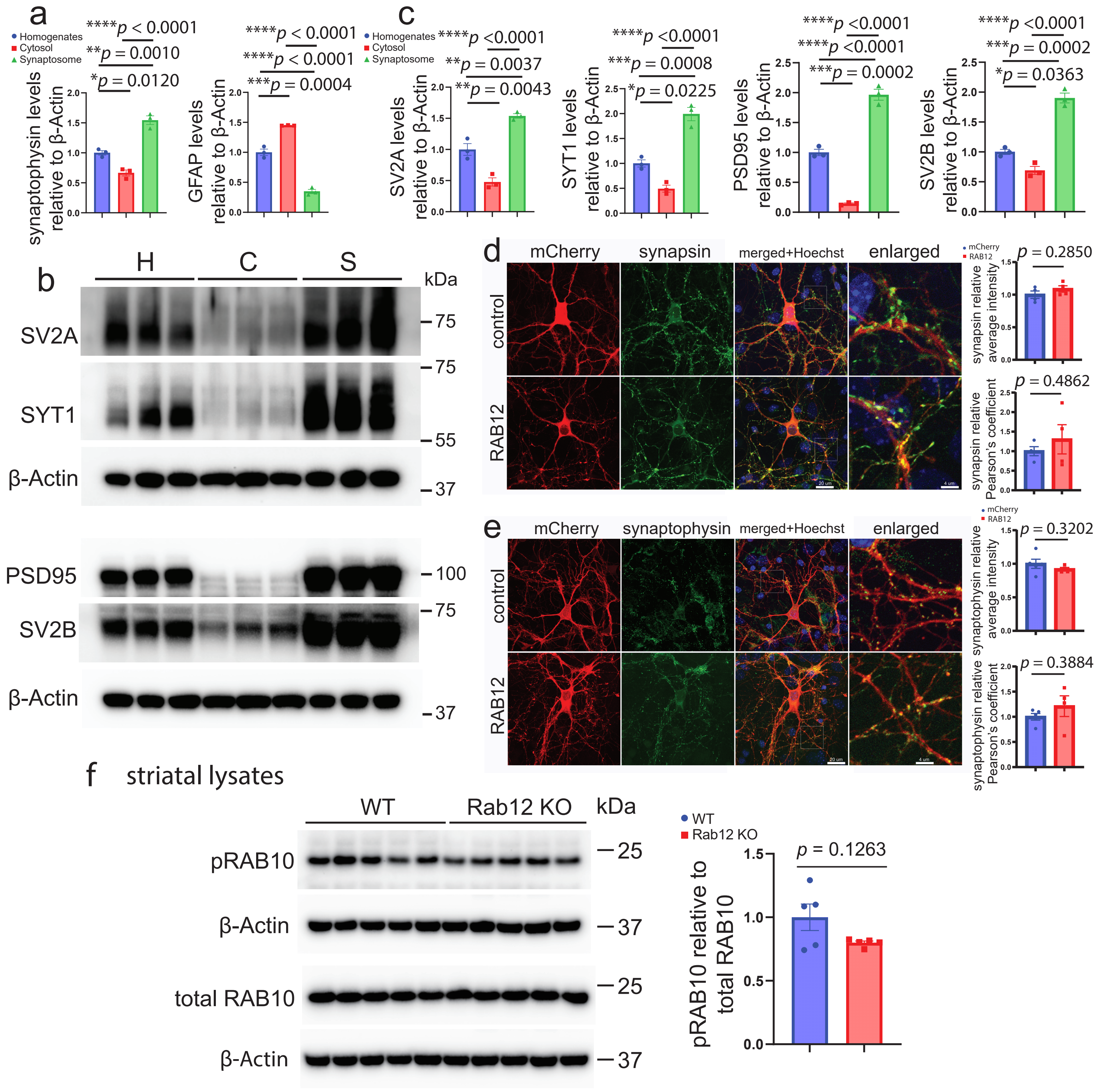

a
Homogenates
Cytosol
Synaptosome
****p < 0.0001
**p = 0.0010
*p = 0.0120
synaptophysin levels relative to β-Actin
****p < 0.0001
****p < 0.0001
***p = 0.0004
GFAP levels relative to β-Actin
c
****p <0.0001
**p = 0.0037
**p = 0.0043
SV2A levels relative to β-Actin
****p <0.0001
***p = 0.0008
*p = 0.0225
SYT1 levels relative to β-Actin
****p <0.0001
****p <0.0001
***p = 0.0002
PSD95 levels relative to β-Actin
****p <0.0001
***p = 0.0002
*p = 0.0363
SV2B levels relative to β-Actin
b
H
C
S
kDa
SV2A
SYT1
β-Actin
PSD95
SV2B
β-Actin
75
75
55
37
100
75
37
d
mCherry
synapsin
merged+Hoechst
enlarged
control
RAB12
mCherry
RAB12
p = 0.2850
synapsin relative average intensity
p = 0.4862
synapsin relative Pearson's coefficient
e
mCherry
synaptophysin
merged+Hoechst
enlarged
control
RAB12
p = 0.3202
synaptophysin relative average intensity
p = 0.3884
synaptophysin relative Pearson's coefficient
f
striatal lysates
WT
Rab12 KO
kDa
pRAB10
β-Actin
total RAB10
β-Actin
25
37
25
37
WT
Rab12 KO
p = 0.1263
pRAB10 relative to total RAB10

Fig. S2 | RAB12 localization in neuronal synapses in support of Fig. 4.

a Quantification of synaptophysin and GFAP levels in Fig. 4a. **b**-**c** The presynaptic markers SV2A, synaptotagmin-1 (SYT1), and SV2B, as well as the postsynaptic marker PSD95, were measured in homogenates (H), cytosol (C), and synaptosome (S) fractions from mouse cortex by Western blot (n = 3). Each target protein's band intensity was normalized to the corresponding β-Actin intensity, followed by normalization to the homogenate fraction. Data in (**a**, **c**) are presented as fold changes relative to the homogenate fractions, expressed as mean ± SEM. **d**-**e** Primary neurons were infected with AAVs carrying a synapsin promoter to overexpress mCherry or mCherry-RAB12. Immunofluorescence staining was performed to assess colocalization with the presynaptic markers synapsin ($n_{mCherry} = 4$, $n_{RAB12} = 4$) and synaptophysin ($n_{mCherry} = 5$, $n_{RAB12} = 4$). Quantification of the fluorescent intensity of synapsin and synaptophysin, as well as their Pearson's correlation coefficients with mCherry or mCherry-RAB12, was performed on 15-20 neurons. **f** Western blot analysis of pRAB10 levels in striatal lysates from WT and *Rab12* KO mice. Each data point represents one mouse. Data in (**a**, **c**) were analyzed using the ordinary one-way ANOVA test, whereas data in (**d**-**f**) were analyzed using the Welch's t-test. Error bars represent the SEM.

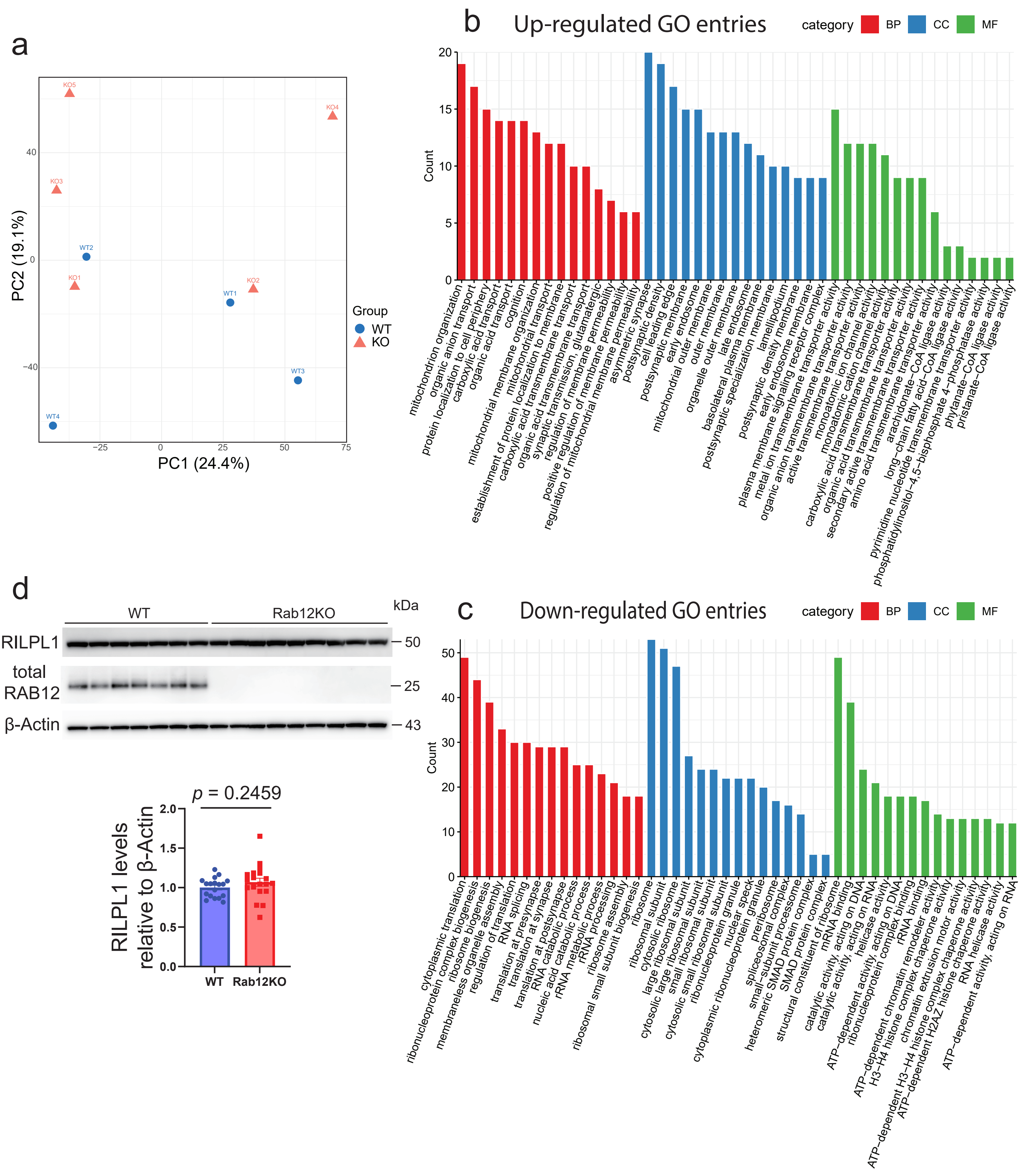

a
PC2 (19.1%)
PC1 (24.4%)
Group
WT
KO
KO5
KO4
KO3
WT2
KO1
KO2
WT1
WT3
WT4
b
Up-regulated GO entries
category
BP
CC
MF
Count
mitochondrion organization
organic anion transport
protein localization to cell periphery
carboxylic acid transport
organic acid transport
cognition
mitochondrial membrane organization
mitochondrial transport
establishment of protein localization to membrane
carboxylic acid transmembrane transport
organic acid transmembrane transport
synaptic transmission, glutamatergic
regulation of membrane permeability
positive regulation of membrane permeability
regulation of mitochondrial membrane permeability
asymmetric synapse
postsynaptic density
cell leading edge
postsynaptic membrane
early endosome
mitochondrial outer membrane
outer membrane
organelle outer membrane
late endosome
basolateral plasma membrane
postsynaptic specialization membrane
lamellipodium
postsynaptic density membrane
early endosome membrane
plasma membrane signaling receptor complex
metal ion transmembrane transporter activity
organic anion transmembrane transporter activity
active transmembrane transporter activity
monoatomic ion channel activity
monoatomic cation channel activity
carboxylic acid transmembrane transporter activity
organic acid transmembrane transporter activity
secondary active transmembrane transporter activity
amino acid transmembrane transporter activity
arachidonate-CoA ligase activity
long-chain fatty acid-CoA ligase activity
pyrimidine nucleotide transmembrane transporter activity
phosphatidylinositol-4,5-bisphosphate 4-phosphatase activity
phytanate-CoA ligase activity
pristanate-CoA ligase activity
c
Down-regulated GO entries
category
BP
CC
MF
Count
cytoplasmic translation
ribonucleoprotein complex biogenesis
ribosome biogenesis
membraneless organelle assembly
regulation of translation
RNA splicing
translation at presynapse
translation at synapse
translation at postsynapse
RNA catabolic process
nucleic acid catabolic process
rRNA metabolic process
rRNA processing
ribosome assembly
ribosomal small subunit biogenesis
ribosome
ribosomal subunit
cytosolic ribosome
large ribosomal subunit
cytosolic large ribosomal subunit
small ribosomal subunit
cytosolic small ribosomal subunit
ribonucleoprotein granule
nuclear speck
cytoplasmic ribonucleoprotein granule
preribosome
spliceosomal complex
small-subunit processome
heteromeric SMAD protein complex
SMAD protein complex
structural constituent of ribosome
mRNA binding
catalytic activity, acting on DNA
catalytic activity, acting on RNA
helicase activity
ATP-dependent activity, acting on DNA
ribonucleoprotein complex binding
rRNA binding
ATP-dependent chromatin remodeler activity
H3-H4 histone complex chaperone activity
chromatin extrusion motor activity
ATP-dependent H3-H4 histone complex chaperone activity
ATP-dependent H2AZ histone chaperone activity
RNA helicase activity
ATP-dependent activity, acting on RNA
d
WT
Rab12KO
kDa
RILPL1
total RAB12
β-Actin
50
25
43
p = 0.2459
RILPL1 levels relative to β-Actin
WT
Rab12KO

Fig. S3 | Proteomic profiling of striatal synaptosomes from *Rab12* KO mice in support of Fig. 5. a Principal component analysis (PCA) of WT and *Rab12* KO synaptosome proteomic datasets. **b**-**c** Gene Ontology (GO) analysis of upregulated (**b**) and downregulated (**c**) DEPs to identify enriched biological processes (BP), cellular components (CC), and molecular functions (MF). The top 10 GO terms in each category are shown ($n_{WT} = 4$, $n_{Rab12KO} = 5$). Entries with $p < 0.05$ and $q < 0.05$ were considered significant. **d** Western blot analysis of RILPL1 in striatal synaptosomes from WT and *Rab12* KO mice ($n_{WT} = 18$, $n_{Rab12KO} = 19$). The Welch's t-test was used for statistical analysis. Error bars represent the SEM.

Fig. 4a

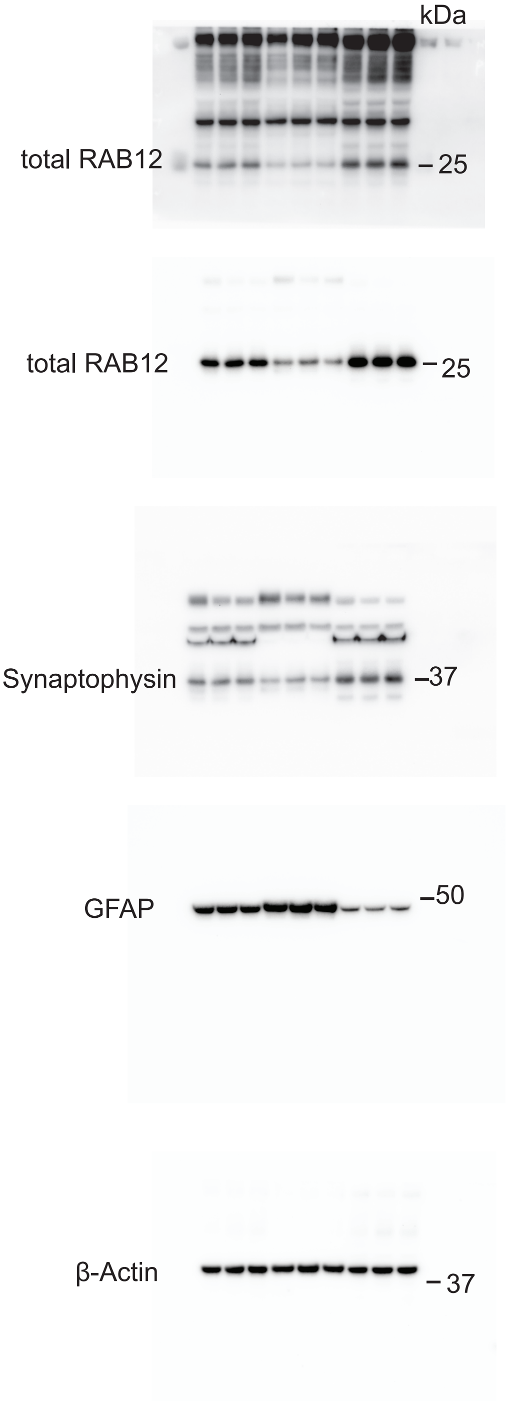

Fig. 4c

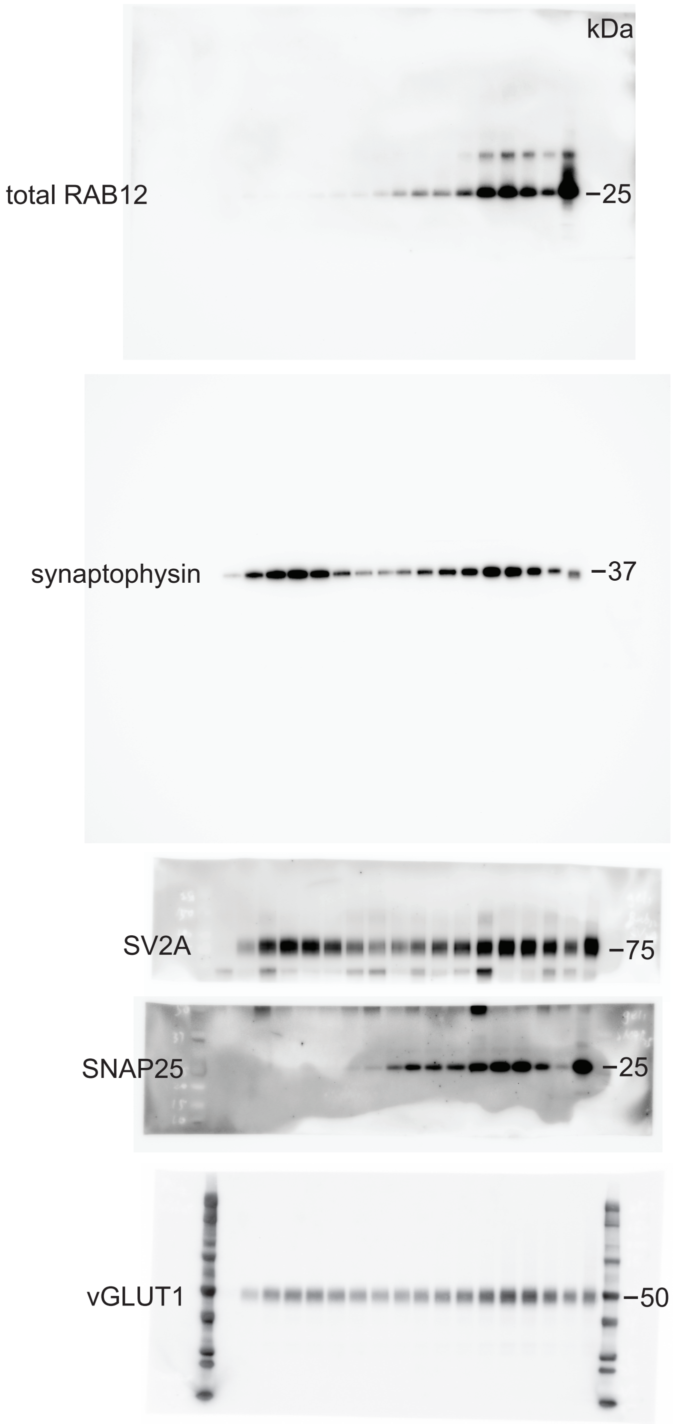

Fig. 4d

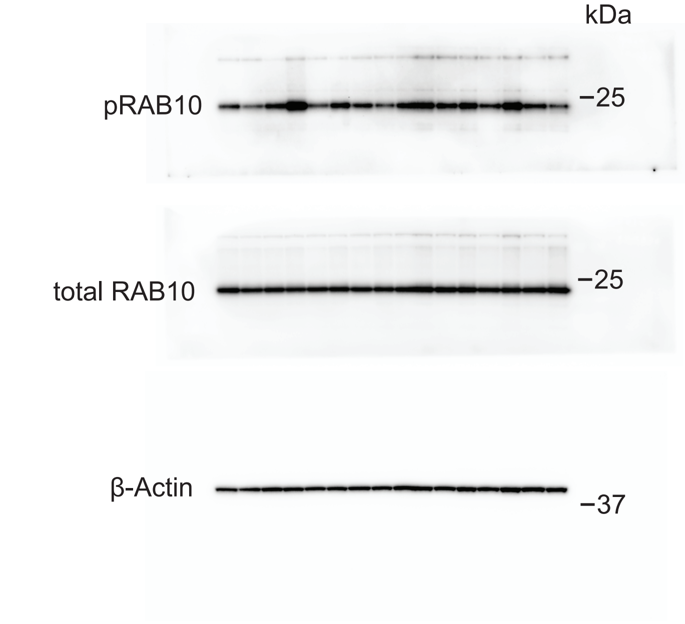

Fig. 5d

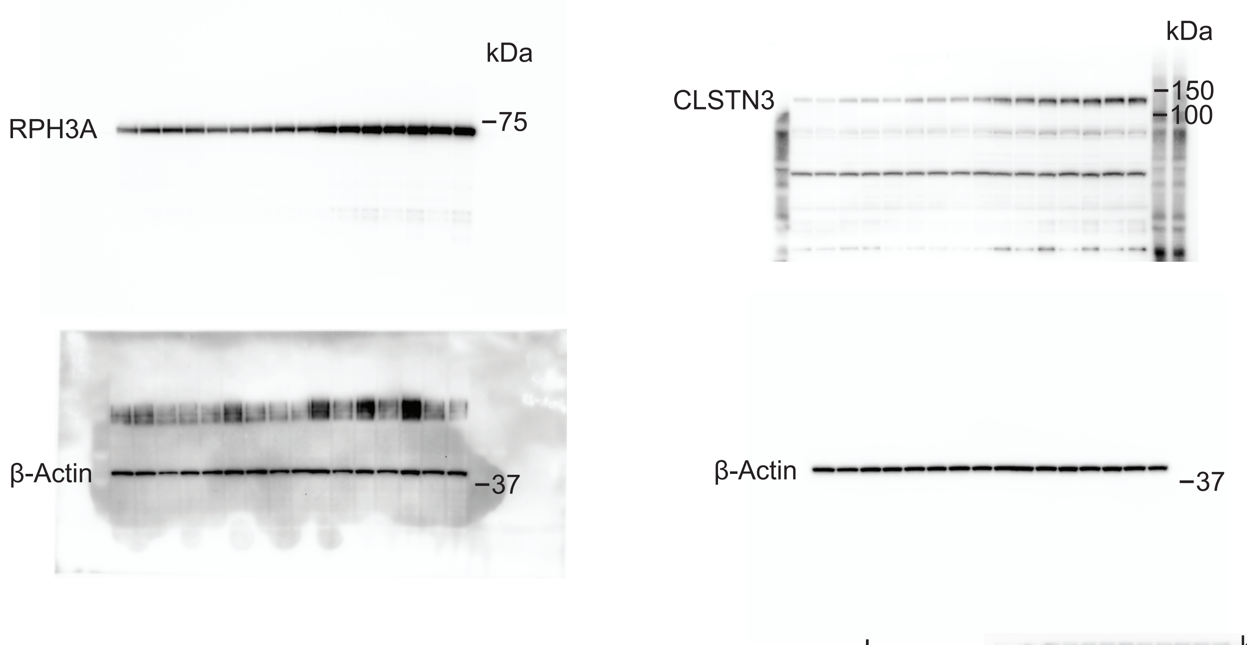

Fig. S2b

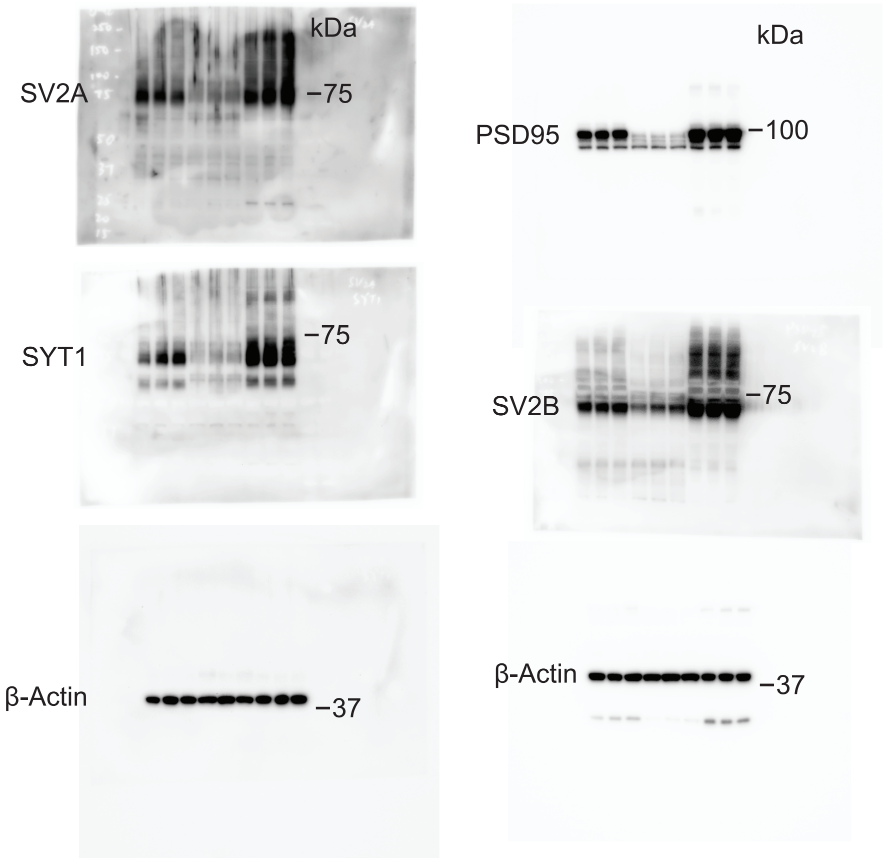

Fig. S2f

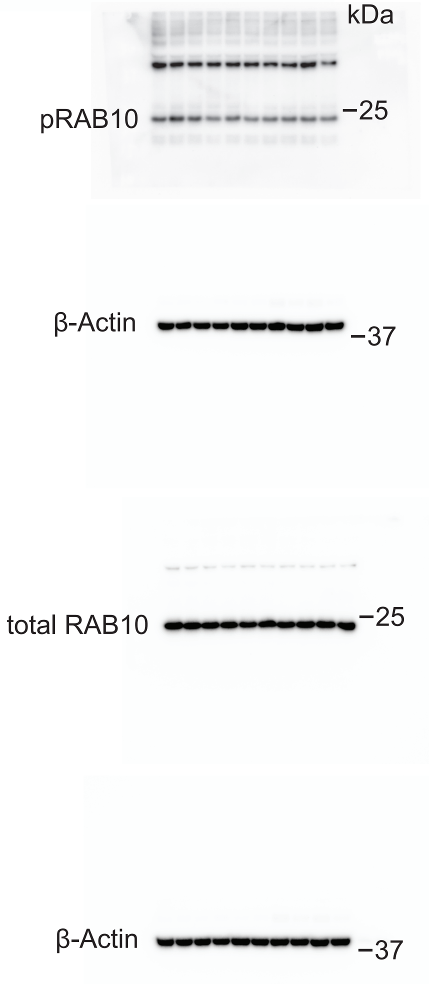

Fig. S3d

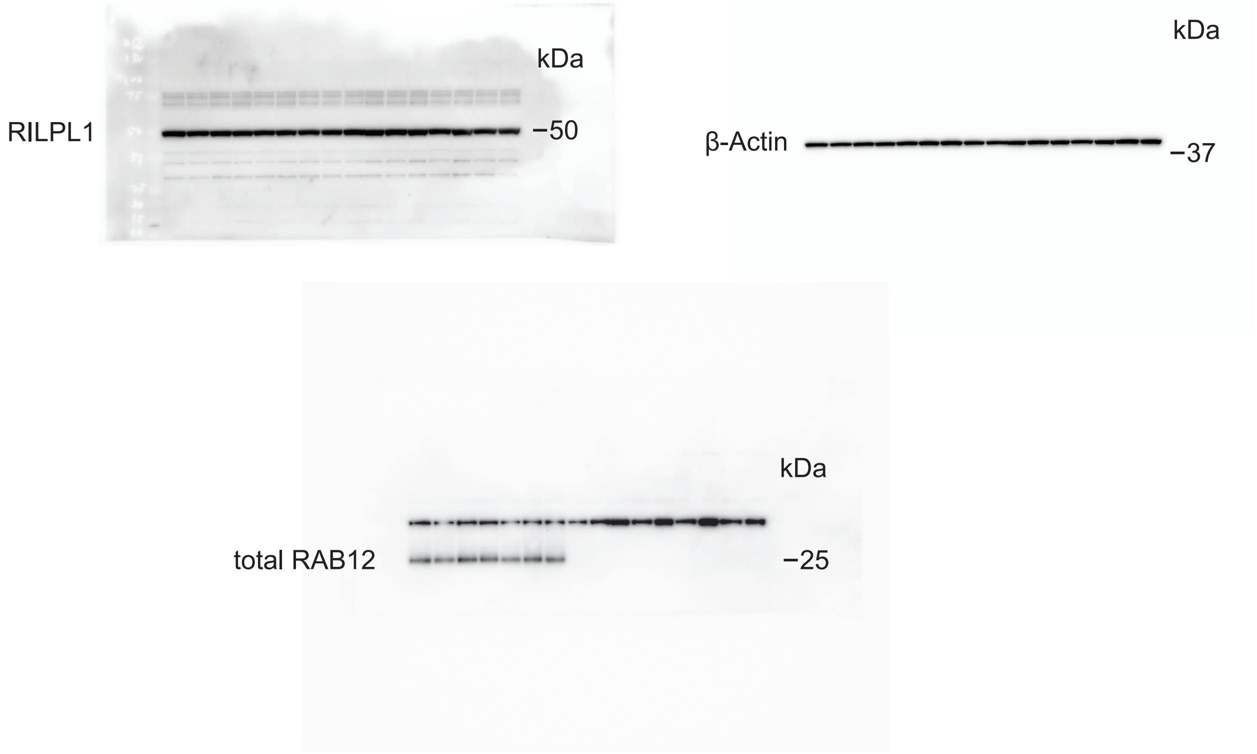

Fig. S4 | Uncropped Western blot images

Supplementary Data

Data S1. Proteomic data of striatal synaptosomes from *Rab12* KO mice.

Data S2. Gene Ontology (GO) biological process analysis of upregulated differentially expressed proteins from striatal synaptosomal proteomics of *Rab12* KO mice.

Data S3. GO cellular component analysis of upregulated differentially expressed proteins from striatal synaptosomal proteomics of *Rab12* KO mice.

Data S4. GO molecular function analysis of upregulated differentially expressed proteins from striatal synaptosomal proteomics of *Rab12* KO mice.

Data S5. SynGO-based Gene Set Enrichment Analysis (GSEA) analysis of proteomic data from striatal synaptosomes of *Rab12* KO mice.

Data S6. GO biological process analysis of downregulated differentially expressed proteins from striatal synaptosomal proteomics of *Rab12* KO mice.

Data S7. GO cellular component analysis of downregulated differentially expressed proteins from striatal synaptosomal proteomics of *Rab12* KO mice.

Data S8. GO molecular function analysis of downregulated differentially expressed proteins from striatal synaptosomal proteomics of *Rab12* KO mice.
